# Reducing Stress and Anxiety in Caregivers of Lung Transplant Patients: Benefits of Mindfulness Meditation

**Published:** 2014-05-01

**Authors:** J. Haines, K. C. Spadaro, J. Choi, L. A. Hoffman, A. M. Blazeck

**Affiliations:** 1 *Department of Acute/Tertiary Care, School of Nursing, University of Pittsburgh, Pittsburgh, USA,*; 2 *School of Nursing, Chatham University, Pittsburgh, USA*

**Keywords:** Caregiver, Mindfulness, Meditation, Lung transplant, Stress, Anxiety

## Abstract

Background: Caregivers are a vital resource in the care of transplant candidates or recipients. However, few strategies have been tested that attempt to decrease the stress and anxiety they commonly encounter.

Objective: To test the feasibility of using mindfulness-based stress reduction (MBSR) techniques to decrease stress and anxiety in caregivers of lung transplant candidates/recipients who required admission to an acute care facility.

Methods: 30 caregivers of lung transplant candidates/recipients were recruited during hospitalization of their significant other. Each completed the perceived stress scale (PSS) and state trait anxiety inventory (STAI) before and 4 weeks after receiving a DVD that demonstrated MBSR techniques. Participants were asked to practice MBSR techniques for 5–15 min a day for 4 weeks.

Results: The participants had a mean±SD age of 55.6±13.6 years; 77% of participants were female and 93% Caucasian. The mean PSS and STAI (trait and anxiety) scores of caregivers were higher than population norms pre- and post-intervention. Scores for caregivers who stated they watched the entire DVD and practiced MBSR techniques as requested (n=15) decreased significantly from pre- to post-testing for perceived stress (p=0.001), state anxiety (p=0.003) and trait anxiety (p=0.006). Scores for those who watched some or none of the DVD (n=15) did not change significantly.

Conclusion: Caregivers can benefit from stress reduction techniques using MBSR.

## INTRODUCTION

Lung transplantation has become a commonly used option for patients with end-stage pulmonary disease with over 24,000 lung transplantations performed in the USA [1, 2]. Patients and caregivers undergo substantial physical and emotional stress during the process of transplantation [3-6]. Prior to the transplantation, both parties must cope with issues arising as a result of the decline in the patient’s condition [7]. When the evaluation process begins, both the patient and caregiver must travel to the transplant center, which is often far from home. In addition to the cost of travel, caregivers may be required to remain at the evaluation site for the time required to complete the pre-transplantation assessment process [8]. Because the number of candidates greatly outnumbers available organs, the wait period prior to transplantation can be long. The post-transplantation period can also be challenging. Lung recipients have the highest rate of rejection and the shortest survival of all organ transplants [9, 10]. Multiple hospital readmissions are common among lung recipients and worsening of the patient’s condition can escalate caregiver distress [11]. 

Previous studies suggest that caregivers of patients who are critically ill experience a high level of stress, anxiety, and depression [12, 13]. Lung transplant candidates/recipients are admitted to an ICU immediately after surgery and may be admitted to a clinical unit or ICU during subsequent readmissions. With these and other hospitalizations, caregivers face multiple stressors. The need for any hospitalization signals deterioration in health status and possible rejection. In a study that used qualitative methodology to explore the implications of chronic rejection after lung transplantation, caregivers reported overwhelming emotions and concern, equating the onset of rejection with mortality [11]. Clinicians were reluctant to discuss prognosis with caregivers despite evident deterioration. This reluctance, while understandable, has the potential to further add to caregiver stress and anxiety. 

There is evidence that the stress of providing care can negatively impact the well-being of patient and caregiver. Myaskovsky, *et al* [7], studied 114 lung transplant candidates and their caregivers and found that better patient quality of life was associated with better caregiver quality of life. This linkage between patient and caregiver quality of life was supported in second study conducted by the same group [10]. In contrast, a study that surveyed 621 primary caregivers of solid organ transplant candidates (lung, liver, heart, kidney) reported that they were generally well adjusted, with few reporting depressive symptoms (17%) or anxiety (13%) [8]. However, the study involved the caregivers living at home during the pre-transplant wait period. 

Mindfulness-based stress reduction (MBSR), developed in 1990 by John Kabat-Zinn [14], is a program of daily meditation and gentle stretching exercises designed to refocus the body’s response to stressors outside of one’s control. The goal of MBSR is to empower individuals to respond consciously, rather than automatically, to circumstances [14]. The basic component of MBSR begins with the person focusing their awareness on the sensation of breathing. Teaching the person to be conscious of their breath is then incorporated into the four formal practices of MBSR—the body scan, sitting meditation, mindful movement, and mindful eating. During the body scan, participants breathe consciously, slowly and deeply while focusing their attention on scanning their body from heel to head. This self-awareness promotes locating, identifying, and releasing any tension present. During sitting meditation, participants focus on slow, deep breathing and concentrate on relaxation. Mindful movement involves gentle stretching exercises performed slowly with the rhythm of the breath paired with body movement. In mindful eating, participants breathe slowly using all of their senses, *ie*, sight, touch, smell, hearing, and taste, to experience their food, a process designed to enable them to enjoy eating rather rushing to finish. Prior studies conducted to assess the effect of MBSR on health outcomes, report that use of MBSR reduces pain, anxiety, and depression in various patient populations, *eg*, patients with fibromyalgia, irritable bowel syndrome, and cancer [14-20]. 

Three studies were identified that tested the ability of MBSR to reduce symptoms in transplant patients [21-23]. Results demonstrated improvement in depression, sleep, and anxiety, with some benefits lasting over a year. We were unable to identify any studies focusing on use of MBSR in caregivers of lung transplant candidates/recipients. Because caregivers of lung transplant candidates/recipients who require hospitalization often spend long periods at the bedside or away from family, we reasoned that MBSR might be beneficial in reducing their stress and anxiety. Therefore, we conducted this study to test the feasibility of using MBSR techniques to decrease stress and anxiety in caregivers of lung transplant patients. 

## MATERIALS AND METHODS

In a pre-test/post-test design, we recruited 30 caregivers of patients admitted to an acute care lung transplant unit as a consequence of deterioration in their health status. The inclusion criteria were: 1) being a primary caregiver for a lung transplant candidate or recipient; 2) age between 30 and 70 years (the common range for recipients at this institution); 3) being able to speak, read, and write English (the DVD and questionnaires were in English); 4) no neurological or hearing deficits (required to use the DVD); and 5) having access to a DVD player. The project received Institutional Review Board approval and all participants provided informed consent. 

MBSR techniques were demonstrated via a DVD developed with guidance from a member of the research team (KS) with training in these techniques. The 60-min DVD included the following sections: introduction (8 min), practice of mindfulness (7 min), demonstration of focusing on the breath (10 min), stress and its effect on the body (7 min), the body scan (20 min), mindful eating (3 min) and sitting meditation (5 min). We elected not to include yoga exercises because the majority of likely participants were away from home without access to a mat to practice this aspect of MBSR. Each caregiver was asked to watch the DVD and practice the exercises for 5–15 min a day for four weeks in their home or place of residence (*eg*, family house). 

Demographic data (age, gender, race, education, *etc*) were collected using a researcher-developed questionnaire. Caregivers also provided data regarding time since transplant, time on wait list and prior experience using any techniques to reduce stress or anxiety. 

The perceived stress scale (PSS) has been used in studies assessing the stressfulness of situations, outcome of interventions designed to reduce stress, and the association between physical disorders and psychological stress [24-28]. Items in the PSS are designed to appraise how unpredictable, uncontrollable, and overloaded respondents find their lives and how confident they feel regarding their ability to manage personal problems [24]. There are three versions of the scale with 4, 10, or 14 items. The 10-item version was used since it has demonstrated maximum reliability [24]. The tool’s Cronbach’s alpha ranges from 0.84 to 0.86 [28]. The test scores range from 0 to 40, with higher scores indicating greater stress. Completion of the test requires approximately 10 min.

The state trait anxiety inventory (STAI) is the leading measure of personal anxiety worldwide [29]. The tool consists of 40 items which include separate measures of state and trait anxiety. State anxiety assesses how the individual completing the survey feels at the present moment, and trait anxiety is a measure of general stress. Measures of internal consistency for the STAI average >0.89, and test-retest reliability are high [29]. Total scores range from 20 to 80, with higher scores indicating increased levels of anxiety. Completion of the test requires approximately 10 min. 

Participants completed the STAI and PSS during an educational session about use of the DVD at the time of consent and after completing the 4-week intervention. They were given the option of face to face, telephone or mailed questionnaires, depending on their preference.

Statistical analysis

Data were analyzed using SPSS^®^ for Windows^®^ ver 20 (SPSS Inc, Chicago, IL, USA). Continuous variables were reported as mean±SD; categorical variables were reported as frequency and proportions. To examine change in study measures, we formed two groups *post hoc—*participants who stated they watched the entire DVD and participated in the exercises (n=15) and those who stated they watched some or none of the DVD (n=15). *Student’s t* test for independent samples and χ^2^ test were used to compare demographic characteristics between the two groups. One-way analysis of variance (ANOVA) was used to examine differences in stress and anxiety as a function of time (pre- *vs* post-intervention) and group membership (watched all *vs* watched some/none). The partial *ƞ*^2^ was used to report the effect sizes, where 0.01 was considered “small,” 0.06 “moderate,” and 0.14 was considered “large” effects. A two-tailed p value <0.05 was considered statistically significant. 

## RESULTS

Of 39 caregivers approached, 30 agreed to participate. Refusal primarily related to lack of time or interest. The mean±SD age of participants was 55.6±13.6 years. Of 30 participants, 77% were female and 93% were Caucasian (Table 1). Half were employed full or part time. All had at least a 9^th^ grade education; one-third had earned college or graduate degrees. The majority (70%) were from Pennsylvania. Of the patients, 28 were lung transplant recipients and two were transplant candidates. The median time since transplantation was 30 (range: 0–120) months. Most caregivers had not participated in a formal stress reduction program. Previous interventions used to reduce stress included prayer, Christian music, humor, deep breathing, yoga, meditation, visual imagery, acupuncture, and walking. 

**Table 1:** Demographic characteristics of participants. Values are either mean±SD or frequency (%).

Characteristic	Watched All DVD (n=15)	Watched Some or None of DVD (n=15)	p value
Caregiver			
Age (yrs)	50.5±13.7	60.7±11.8	0.04
Gender			
Female Male	13 (87%) 2 (13%)	10 (67%) 5 (33%)	0.390
Race			
Caucasian African-American	15 (100%) 0 (0%)	13 (87%) 2 (13%)	0.483
Marital status			
Married Widowed, separated, divorced	13 (87%) 2 (13%)	13 (87%) 2 (13%)	0.701
Education			
High school or GED Technical school or some college College graduate or higher	1 (7%) 10 (67%) 4 (27%)	4 (27%) 5 (33%) 6 (40%)	0.145
Employed			
Full or part time Not working	6 (40%) 9 (60%)	9 (60%) 6 (40%)	0.466
Use stress reduction			
Yes No	3 (20%) 12 (80%)	3 (20%) 12 (80%)	1.00
Transplant Candidate/Recipient			
Time on wait list (days)	44.3±81.5	108.4±131.2	0.316
Time since transplant (months)	37.2±33.8	22.2±29.0	0.202

While all agreed to participate, 15 self-reported they watched the entire DVD and performed the exercises; six self-reported they watched some and nine watched none of the DVD. Reasons stated for not watching the DVD included lack of time and deterioration of the patient’s condition resulting in increased anxiety and reshuffling of priorities. There were no significant differences in demographic characteristics between caregivers who watched the entire DVD and those who watched some or none, except age of the caregivers—those who watched the entire DVD were significantly (p=0.04) younger than those who watched some or none of the DVD. 

Perceived stress

Tests for main effects between groups revealed no significant (p=0.666, partial *η*^2^ =0.007) difference between the group who watched the entire DVD and the group who watched some or none. The main effects for the change in PSS scores from pre- to post-intervention was statistically significant (p=0.001, partial *η*^2^ =0.33). The interaction effect between DVD viewing and pre/post changes was also significant (p=0.02, partial *η*^2^ =0.19). *Post hoc* analysis revealed that the decrease in PSS scores from pre-intervention (22.13±7.14) to post-intervention (13.93±5.28) was statistically significant (p=0.001) for the group who watched the entire DVD. In caregivers who watched some or none of the DVD, the change from pre-intervention (19.80±8.02) to post-intervention (18.27±8.09) was not statistically significant (p=0.35). 

State anxiety

As with PSS, tests for the main effects between groups revealed no significant difference (p=0.279, partial *η*^2^ =0.042) between the caregiver group who watched the entire DVD and the group who watched some or none. Overall, there was a significant (p=0.001, partial *ƞ*^2^ =0.32) decrease in state anxiety from pre-intervention to post-intervention averaged across the DVD groups. Again, there was a significant (p=0.05, partial *ƞ*^2^ =0.14) interaction between DVD groups and pre/post changes. *Post hoc* analysis found that for caregivers who watched the entire DVD, state anxiety decreased significantly (p=0.003) from pre-intervention (46.20±13.80) to post-intervention (36.73±8.10). In caregivers who watched some or none of the DVD, the change from pre-intervention (47.60±14.46) to post-intervention was not statistically significant (p=0.22).

Trait anxiety

Similar findings were seen for trait anxiety. There were no significant (p=0.820, partial *ƞ*^2^ =0.002) differences between the “all” *vs* “some or none” DVD groups. There was a significant (p=0.004. partial *ƞ*^2^ =0.26) decrease in trait anxiety from pre-intervention to post-intervention averaged across the group. The interaction effect between DVD group and pre/post change was not significant (p=0.053, *ƞ*^2^ =0.127). However, *post hoc* analysis revealed the same pattern of outcomes. In caregivers who watched the entire DVD, trait anxiety decreased significantly (p=0.006) from pre-intervention (44.27±14.11) to post-intervention (34.07±7.90) while the change was not statistically significant (p=0.38) from pre-intervention (41.07±11.32) to post-intervention (38.87±9.64) in those who watched some or none.

Comparison with norms

In addition, we compared the mean PSS scores with norms established by Cohen, which were 12.1 for males and 13.7 for females [22]. The mean PSS scores were higher than population norms at pre-intervention (20.97±7.56) and post-intervention (16.10±7.07). When compared with age and gender-matched norms established by Spielberger (state anxiety: 34.51 for males and 32.20 for females; trait anxiety: 33.86 for males and 31.79 for females) [27], similar findings were seen. The mean state anxiety scores were higher than population norms at pre-intervention (46.90±13.90) and post-intervention (40.90±12.07), as were mean trait anxiety scores (42.67±12.68 *vs* 36.47±9.00, respectively).

## DISCUSSION

Although MBSR has been tested in other patient populations, to the best of our knowledge, this is the first study where it was used with caregivers of lung transplant patients. Our findings provide preliminary evidence that this intervention can decrease stress and anxiety in caregivers who watched the entire video and practiced the concepts demonstrated. Because our study did not include a control group, it is possible that the changes observed were resulted from increased attention given to the participants. This appears unlikely as those who watched a portion or none of the DVD did not experience a significant change in stress or anxiety. Future studies using a randomized design are needed to confirm this finding. 

Caregivers of lung transplant candidates/recipients are known to experience stress that can escalate during hospitalization of their family member. In our participants, the mean PSS scores at pre-intervention were higher than the population norms provided by Cohen [24]. Although our intervention was successful in reducing PSS scores, the mean scores at post-intervention remained above norms, reinforcing the high stress level experienced by our participants. Similar findings were reported for mean scores of both trait and state anxiety, which were also higher than age and gender-matched norms. 

Our results are consistent with prior studies that tested the ability of MBSR to decrease stress and anxiety. Hoffman, *et al* [15], studied 229 breast cancer patients comparing MBSR to the standard care. Those using MBSR showed decreased mood disturbance and lower levels of anxiety depression, anger, fatigue and confusion. A study of 172 breast cancer patients found MBSR improved quality of life and coping outcomes [16]. Lengacher, *et al* [17], found that breast cancer patients learning MBSR reported deceased depressive symptoms, anxiety and fear of recurrence in addition to improved energy and physical role functioning. Speca, *et al* [18], reported a decrease in depression, anxiety, anger, confusion, stress, irritability, cardiac and gastrointestinal symptoms as well as increased vigor when MBSR was compared to usual care in cancer patients. Fibromyalgia patients in the MBSR treatment group of 91 participants experienced decreased depression [19]. Gaylord, *et al* [20], reported patients with irritable bowel syndrome experienced decreased bowel symptom severity and improved quality of life. 

Our preliminary results compare favorably with results from studies in other populations that used more complex interventions to decrease stress in caregivers. Examples include a worksite-based internet multimedia program and a six-week disease management program [30, 31]. Both studies used complex interventions that are time-intensive and therefore costly in terms of resources. A major advantage of our approach relates to its minimal cost and use of resources. 

This study had several limitations. While the majority of studied caregivers readily agreed to participate in the study, approximately half did not fully carry out the intervention. This finding was unexpected. We recommend that future studies include a return demonstration of MBSR techniques and self-report log to provide more detail regarding intervention fidelity. Although diaries have known limitations, the data obtained would provide an additional means to estimate use of MBSR. Caregivers were recruited from one institution, sample size was small, and follow-up was relatively short. Future research with a larger sample followed for a longer period is strongly recommended. Finally, recruitment occurred in a clinical unit. While it would be beneficial to recruit caregivers at an earlier, less stressful time, offering MBSR during acute care admission has the advantage of providing support when it is likely most needed and beneficial. 

In summary, our pilot findings suggest that MBSR may have the potential to decrease stress and anxiety in caregivers of lung transplant patients. The DVD was an easy, cost-effective form of instruction that can be used by nursing staff. Those who carried out the intervention as advised experienced a reduction in stress and anxiety. Caregivers are a vital resource and the need for reducing stress and anxiety in this population is substantial. Further study is warranted using a control group and participants followed for a longer time interval. 
